# Poisoning from Chloral Hydrate

**Published:** 1871-04

**Authors:** 


### ARTICLE V.

### *Poisoning from Chloral Hydrate.*

Reports of fatal results from the use of chloral are becoming unpleasantly numerous. Not long ago, we commented on a case recorded by Dr. Needham in the *Journal of Psychological Medicine*, where death followed the administration of this agent under skilled advice and in moderate doses, and there lie before us now the accounts of several other mortal accidents. In the *British Médical Journal* of the 25th ultimo, is given the first instance of poisoning by this drug reported in England, in the case of a clergyman who had acquired the habit of taking chloral at night, commencing with scruple doses. During two months, the dose was gradually increased, until it is estimated that he took fourteen or fifteen drachms in the ten days preceding the 27th of January, on the morning of which latter day he was found dead in his bed. The only abnormal post mortem appearances were increased vascularity of the stomach at its cardiac extremity and along the lesser curvature, with minute submucous extravasated patches of blood in the former situation (the patient had been a sufferer from dyspepsia); a marked congestion of the cerebral membranes, which contained about an ounce of red serum, and presented old adhesions to a small extent on each side of the superior longitudinal sinus; and pallor with softness and friability of the cerebral substance. "There was no increased vascularity in any part except the choroid plexus; no effusion in the ventricles, nor extravasation of blood." These appearances are very different from those observed in the autopsy of Dr. Needham's case, where increased vascularity pervaded the entire brain, the only point of resemblance between the two cases being that in both there was found great congestion of the membranes.

In the *Buffalo Medical and Surgical Journal*, Dr. Holbrook, of Palmer, Mass., reports the death of the wife of a clergyman in that town. This lady took the entire contents of an ounce vial of chloral, from which her husband had taken but three doses, the quantity ingested by her being estimated at over one hundred grains. About twelve hours afterwards, Dr. Holbrook found her in a comatose condition, with contracted and insensible pupils, injected conjunctivæ, livid face, and cold extremities; the heart's action very feeble. Death ensued in about fifteen hours from the time of taking the drug, the patient never recovering consciousness. No autopsy was made. [A recovery from a still larger dose of chloral is reported in the *Medical Times* of Oct. 15, 1870, in the case of a nurse in the Philadelphia Hospital, who took 460 grains in a single draught. Restoration was effected by means of sinapisms to the extremities, vigorous flagellation, and the application of a powerful Faradic current along the spinal column, the phrenic nerves and to the chest.]

The Hartford *Times* recently reported the case of Frederick Ripley, a station-agent at Avon, Conn., for whom chloral was prescribed as a hypnotic by a physician. For about ten days it was taken with pleasant effect; at the end of that time a fresh supply was obtained from a druggist in New Haven, the first dose of which was followed by death in half an hour. Coming from an unprofessional source, the account of the symptoms is of course unsatisfactory, but there seems to be reason to suspect that in this instance some irritant poison was mixed with the chloral.

The San Francisco *Chronicle* of the 7th instant, contains a highly sensational article on the death of a Mr. Hixon, of that city, who, on the recommendation of a friend, took one evening about an ounce of an advertised "elixir of chloral hydrate," and was found dead in bed next morning. In the report of the coroner's examination, no mention is made of the condition of the brain and meninges; but the thoracic and abdominal organs are said to have been healthy, with the exception of slight traces of disease in the kidneys, and a somewhat congested state of the lungs.

When first unfavorable results from the administration of chloral were brought to notice, we were inclined to suspect the purity of the drug; but from a number of analyses published in the *British Medical Journal* it would appear that there is little variation in the samples of different manufacturers, at least as far as the English market is concerned. In six specimens examined, the percentage of chloroform by weight was from 68.11 to 69.61; the percentage of chloral hydrate in the corresponding samples being from 94.33 to 96.41. Nor does it seem, as has been feared, that an alcoholate instead of a hydrate of chloral is often found in commerce. It is, therefore, to physiological rather than to analytical chemistry that we must look for an explanation of many cases of chloral poisoning. Our knowledge of its physiological action is as yet very far from exact, the only established contradictions to its use being in cases of organic cerebral or cardiac disease, with a little heeded caution against its hypodermic injection. Three pharmaceutical points, however, it may be well to bear in mind: Firstly, that chloral hydrate when procurable in hard, transparent crystals is less likely to contain impurities than when in the form of an opaque, amorphous cake; secondly that a simple test of its purity is its property of disengaging chloroform when mixed with an alkaline solution, without alteration of color; thirdly, that if kept in mixture for any length of time, it is apt to undergo decomposition, which renders it either inert or dangerous, and that, consequently, all preparations ready-made for domestic use are to be unhesitatingly condemned.—*Medical Gazette.*

